# Detection of *MC1R* Genetic Variants and Their Association with Coat Color in Asian Goats

**DOI:** 10.3390/ani15142026

**Published:** 2025-07-10

**Authors:** Fuki Kawaguchi, Amane Shaku, Manoj Kumar Shah, Joseph S. Masangkay, Hideyuki Mannen, Shinji Sasazaki

**Affiliations:** 1 Laboratory of Animal Breeding and Genetics, Graduate School of Agricultural Science, Kobe University, Kobe 657-8501, Japan; kawaguchi@koala.kobe-u.ac.jp (F.K.); tunalove0401@gmail.com (A.S.); mannen@kobe-u.ac.jp (H.M.); 2 Nepal Agricultural Research Council, Pakhribas 56800, Nepal; manojnarc@gmail.com; 3 Department of Veterinary Paraclinical Sciences, University of the Philippines, Los Banos 4031, Philippines; jsmasangkay@yahoo.com

**Keywords:** goat, coat color, MC1R, Asia, gene variants

## Abstract

Mammals display a wide range of coat colors, with the melanocortin 1 receptor gene (*MC1R*) playing a pivotal role in the genetic regulation of pigmentation. In this study, we investigated *MC1R* genetic variants in goat populations from four Asian countries to identify genetic factors associated with coat color variation. The complete coding sequence of *MC1R* was analyzed to detect variants and determine genotypes in goats from Nepal (*n* = 122), the Philippines (*n* = 110), Cambodia (*n* = 30), and Kazakhstan (*n* = 30). Seven variants were identified, three of which were novel, while four had been previously reported in other goat populations. Among the novel variants, a frameshift mutation (c.147delG) unique to Nepalese goats was predicted to cause substantial disruption of the MC1R protein structure, suggesting its potential role in coat color determination. Additionally, two known missense variants (c.676A > G and c.801C > G) were inferred to affect MC1R function and may contribute to pigmentation variation. Further investigations with larger sample sizes are warranted to clarify the phenotypic effects of these genotypic variants and to better understand the genetic basis of coat color in goats.

## Simple Summary

In this study, we examined coat color variation in Asian goat populations to elucidate the genetic factors regulating this trait. We specifically investigated the melanocortin 1 receptor gene (*MC1R*), recognized for its critical role in pigmentation, to identify genetic variants and assess their association with coat color phenotypes. Seven variants were detected within the *MC1R* gene across the Asian goat populations analyzed. Notably, a novel frameshift mutation unique to the Nepalese goat population, along with two previously reported nonsynonymous substitutions—also identified in other goat populations—were strongly associated with functional alterations of MC1R, indicating a significant role in coat color determination. These findings enhance our understanding of the molecular mechanisms governing pigmentation in mammals.

## 1. Introduction

Mammals display a diverse range of coat colors, primarily regulated by pigment synthesis processes within melanocytes [1]. In these cells, the melanocortin 1 receptor (MC1R), a seven-transmembrane G protein-coupled receptor, plays a pivotal role. The binding of two distinct ligands to MC1R triggers the production of different types of melanin [2]. When α-melanocyte-stimulating hormone (α-MSH) binds to MC1R, it enhances tyrosinase activity, promoting eumelanin synthesis and resulting in black or brown pigmentation [1,3]. Conversely, binding of the agouti signaling protein inhibits tyrosinase activity, favoring the production of pheomelanin and leading to red, yellow, or lighter coat colors [1,3]. Based on these interactions (Figure S1), genetic variants in the *MC1R* gene are believed to influence ligand-binding affinity and alter the eumelanin-to-pheomelanin ratio, thereby contributing to variations in coat color phenotypes [4].

Numerous genetic variants in the *MC1R* gene influencing coat color have been identified across a wide range of mammalian species. In cattle, for instance, the p.Gly104fs frameshift mutation corresponds to a recessive loss-of-function allele (e), whereas the p. Leu99Pro substitution is linked to a dominant gain-of-function allele (ED), resulting in brown and black coat colors, respectively [5,6,7]. In yaks, two genotypic variants in complete linkage disequilibrium (p. Gln114Lys and p. Ala291Thr) are associated with dominant black nasal pigmentation [8]. In rabbits, the c.304_333del deletion has been identified as a recessive loss-of-function allele (e) connected to brown coat coloration. Similar associations between *MC1R* variants and pigmentation have been reported in many other mammals, including mice [3], humans [9], guinea pigs [10], pigs [11], horses [12], sheep [13], dogs [14,15], foxes [13], bears [16], felids [17], rabbits [18], and pocket mice [19].

In goats, several *MC1R* nucleotide variants have also been identified and linked to variations in coat color. A study of six European breeds reported three missense mutations (p. A81V [c.242C > T], p. F250V [c.748T > G], and p. C267W [c.801C > G]), one silent mutation (c.183C > T), and one nonsense mutation (c.673C > T) within the coding sequence [20]. Among these, c.801C > G and c.673C > T were significantly associated with black and spotted brown coat phenotypes, respectively [20,21]. Additionally, the c.676A > G variant identified by Wu et al. [22] has been associated with brown pigmentation localized to the head and neck regions in Boer goats. Further investigations have expanded to goat populations across Europe and North Africa [23], China [24], and India [25]. Some variants exhibit geographic specificity; for example, the c.764G > A mutation found in Indian goats is thought to impair MC1R’s ligand-binding affinity and downstream signaling, thereby contributing to brown coat coloration [24].

Several studies using mitochondrial DNA sequences and Y-chromosome markers have revealed substantial genetic divergence between goat populations from Europe/North Africa and Asia [26,27]. However, information on *MC1R* genetic variants in Asian goats remains scarce and has been largely confined to a few regions. Therefore, the present study aimed to explore *MC1R* genetic variants in goats from four previously unstudied Asian countries—Nepal, the Philippines, Kazakhstan, and Cambodia—and to evaluate their potential associations with coat color variation.

## 2. Materials and Methods

### 2.1. Samples

**Figure 1:**
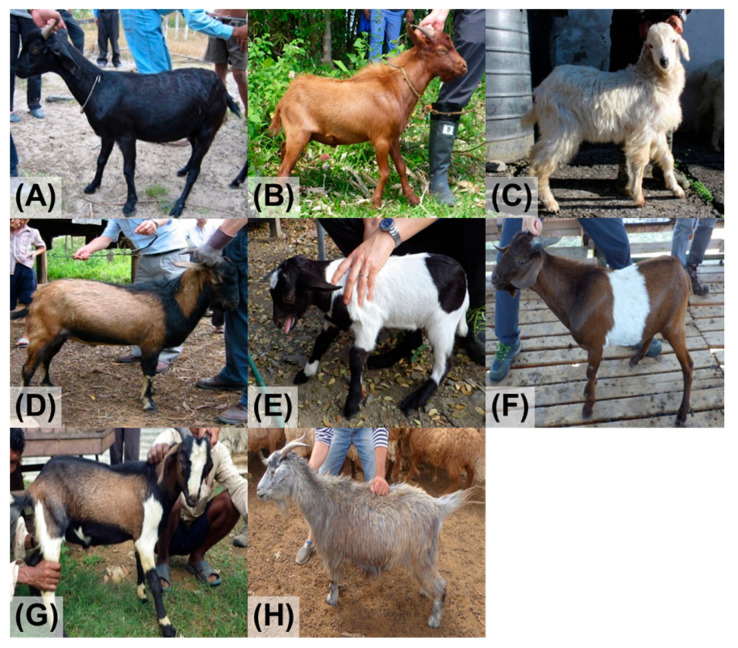
Coat colors in Asian goats. (**A**) black, (**B**) brown, (**C**) white, (**D**) black/brown, (**E**) black/white, (**F**) brown/white, (**G**) black/brown/white, (**H**) roan (black).

In this study, we collected DNA samples from a total of 292 native goats exhibiting diverse coat colors (Figure 1). The samples were obtained from Nepal (*n* = 122) between 2017 and 2018, from the Philippines (*n* = 110) between 2018 and 2019, from Cambodia (*n* = 30) between 2003 and 2004, and from Kazakhstan (*n* = 30) between 2013 and 2014 (Table S1). These animals did not have specific coat color distributions in each country. DNA extraction was performed using the standard phenol–chloroform method on blood samples collected from each goat.

### 2.2. Sequencing

To identify and genotype genetic variants, we sequenced the full-length coding sequence (CDS) of the *MC1R* gene, which was composed of only one exon, in a total of 120 goats, with 30 individuals selected from each of the four countries. During animal selection, we ensured variation in the collection sites and coat colors (black, brown, white, black/brown, black/white, brown/white, black/brown/white, and roan). Additionally, country-specific variants identified during sequencing were genotyped across all sampled individuals from each respective country (Nepal, *n* = 92; Philippines, *n* = 80, additionally). PCR amplification was performed using forward (GAAGAGCGACACTGCACCCA) and reverse primers (TGCAGAATGAGGAGGCTGGG), designed from the GenBank sequence [NC_030825.1] using OLIGO 7.41. Following PCR amplification, the products were purified with ExoSAP-IT^®^ (Affymetrix, Inc., Santa Clara, CA, USA). We then carried out standard double-stranded DNA cycle sequencing using approximately 20 ng of purified product and the BigDye^®^ Terminator v3.1 Cycle Sequencing Kit (Applied Biosystems, Tokyo, Japan) on an ABI PRISM^®^ 3100 Genetic Analyzer (Applied Biosystems, Tokyo, Japan). Both forward and reverse primers were employed to sequence the full-length CDS from both directions. Variant identification was conducted by comparing the sequences of the 120 goats using MEGA X software.

### 2.3. Statistical Analysis

We categorized the 120 sequenced goats into three groups based on their coat colors: black, brown, and white (Table S1). Due to the prevalence of mixed coat colors, the goats were classified into these groups according to specific criteria. Goats exhibiting black in their coat color (black, black/brown, black/brown/white, or roan) were categorized under black, regardless of the presence of other colors. Goats displaying brown without black (brown, brown/white) were assigned to the brown group. Those with exclusively white coats (white) were placed in the white group. A chi-square test was conducted to determine statistically significant differences in allele frequencies among the groups, thereby assessing the association between coat colors and the three missense variants identified in this study.

### 2.4. In Silico Protein Structural Analysis

The DNA sequence of the *MC1R* gene was obtained in FASTA format (FM212940.1) from the NCBI database. As this sequence represented the wild-type, we manually modified it to generate mutant-type sequences. These wild-type and mutant sequences were then submitted to the SWISS-MODEL online tool (https://swissmodel.expasy.org/, accessed on 1 May 2025) for the prediction of protein structure models. The resulting models were downloaded as.pdb files. Subsequently, we used PyMOL v.2.2.0 software to visualize these models. We observed differences in the estimated protein structures between wild and mutant types.

### 2.5. Sequence Alignment

The reference nucleotide and amino acid sequences of the *MC1R* gene from goats, cattle, and sheep were obtained from the NCBI database (goat: MT186766.1 and QMU84787.1; cattle: NM_174108.2 and NP_776533.1; sheep: NM_001282528.1 and NP_001269457.1). These sequences were aligned and analyzed using Clustal W to assess sequence homology among the three species.

## 3. Results

### 3.1. Genetic Variant Discovery and Characterization

**Figure 2:**
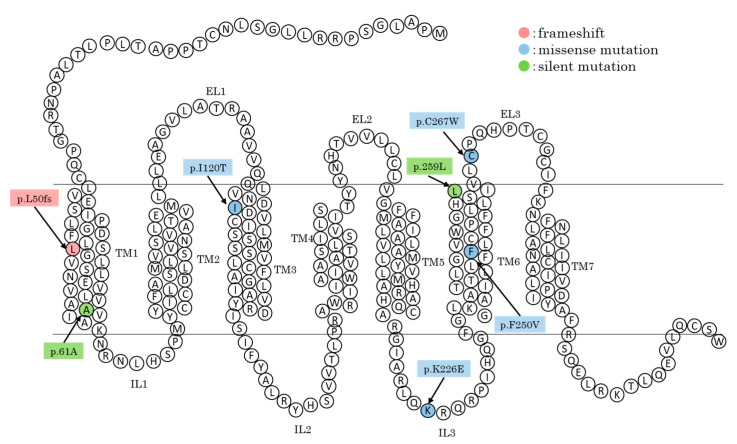
Secondary structure of MC1R with variant locations. TM: transmembrane domain, EL: extracellular loop, IL: intracellular loop.

Through variant screening in this study, a total of seven variants were identified across the full CDS of the *MC1R* gene (Table 1). Four of them were located on c.359, c.676, 748, and c.801 and caused amino acid substitutions, p.I120T, p.K226E, p.F250V, and p.C257W, respectively. Two of them were synonymous variants on c.183 (p.61A) and c.775 (p.259L). The last was a deletion of the G allele at 147th nucleotide and caused a frameshift (p.L50fs). Among these, four nucleotide variants (c.183, c.676, c.748, and c.801) had been previously reported, while three (c.147, c.359, and c.775) were newly discovered in the present study. The three novel SNPs are unique to one country, Nepal or the Philippines, in the context of this research. The allele frequencies and the corresponding positions of these genotypic variants within the MC1R protein are summarized in Table 2 and illustrated in Figure 2. The allele frequencies of major alleles for c.183 and c.748 showed widely range, 0.33–0.75, in each country. That of c.676 was moderate to high, 0.58–0.92. On the other hand, the rest (c.147, c359, c.775, and c.801) showed high major allele frequencies, >0.72, in all countries.

Among the three novel variants, c.147delG, detected exclusively in Nepalese goats, is a frameshift mutation that introduces a premature stop codon at the 51st amino acid. Among all 122 goats sampled from Nepal, 107 were homozygous for the GG genotype with a frequency of 0.83, 12 were heterozygous (G-) with a frequency of 0.13, and 3 were homozygous for the deletion (-- genotype) with a frequency of 0.03 (Table 3). No distinct coat color pattern was observed in goats with the GG or G- genotypes, whereas all three goats carrying the -- genotype exhibited a brown coat color (Figure S2). Additionally, the c.359T > C and c.775C > T variants, identified exclusively in native Philippine goats, were characterized as missense and synonymous mutations, respectively. Among the 110 Philippine goats analyzed, 105 were homozygous for the major allele, 4 were heterozygous, and 1 was homozygous for the minor allele, with complete linkage disequilibrium observed between these two variants (Table 3). No consistent genotype–phenotype association was detected, as goats with the major allele displayed various coat colors, and heterozygous individuals showed several coat color patterns, including brown (two individuals) and black/white or brown/white (one individual each) (Figure S3). Furthermore, protein structural analysis revealed no substantial differences between the c.359T > C alleles (Figure S4). The calculated root mean square deviation (RSMD) value was 0.049, indicating minimal structural alteration due to this nucleotide variant.

### 3.2. Associations Between Variants and Coat Colors

Following previous studies, genotype frequency differences among the three coat color groups in the combined population (*n* = 120) were assessed for the three missense variants (c.676A > G, c.748T > G, and c.801C > G) (Table 4). Statistically significant differences were observed between the black and white coat color groups for all three variants (*p* < 0.05). A trend toward significance was also noted between the black and brown groups (*p* < 0.10). For c.676A > G, a similar trend toward significance was found between the brown and white groups (*p* < 0.10).

### 3.3. Comparison of MC1R Sequences Among Species

Comparison of the *MC1R* gene nucleotide and amino acid sequences among species was performed between goats and other ruminants (cattle and sheep). The CDS nucleotide length was identical across all three species, comprising 954 base pairs. The nucleotide sequence homology was 96.21% between goats and cattle and 98.64% between goats and sheep. Correspondingly, the amino acid sequence homology was 96.12% between goats and cattle and 99.37% between goats and sheep. These high degrees of conservation suggest that the MC1R protein structure is highly preserved across species, allowing the potential functional impacts of allelic variants to be evaluated interspecifically based on their positional context within the gene.

## 4. Discussion

Among the seven genetic variants identified in this study, c.147delG was detected as a novel mutation exclusive to the Nepalese goat population in the context of this research. This variant is a frameshift mutation that substitutes the 50th leucine to trypsin and introduces a premature stop codon at the 51st amino acid of MC1R, likely causing significant disruption to the gene’s function. Consistent with this, all three goats homozygous for the deletion (-- genotype) exhibited a brown coat color. In cattle, the recessive e allele, which induces a frameshift mutation at the 104th amino acid, is known to result in a red coat color [5,6]. Similarly, in dogs and mice, frameshift mutations or premature stop codon insertions affecting the functional domains of MC1R have been associated with brown fur phenotypes [3,14,15]. Based on these findings, it is likely that the c.147delG variant contributes to the brown coat color observed in Nepalese goats. The minor allele frequency of this mutation was low (0.07), and only three goats homozygous for the deletion were identified. Therefore, additional studies involving larger sample sizes are required to more definitively determine the phenotypic impact of this variant.

Additionally, the c.359T > C and c.775C > T variants, identified exclusively in the Philippine goat population in the context of this research, were found to be in complete linkage disequilibrium. The c.775C > T substitution is synonymous and is therefore unlikely to have a significant impact on MC1R function. In contrast, c.359T > C is a missense mutation located within the third transmembrane domain (TM3), resulting in a hydrophobic (I)-to-hydrophilic (T) amino acid substitution. In cattle, a missense mutation within TM3 (p. R142C) has been associated with changes in coat and muzzle color and may influence the subcellular localization of MC1R [28]. Conversely, in sheep, an amino acid substitution at TM3 (p. D121N) was shown to have minimal effect on ligand binding [13]. In the present study, protein structure analysis revealed only minimal structural differences between the alleles, suggesting that the c.359T > C variant is unlikely to exert a substantial effect on MC1R function.

The remaining four variants—c.183C > T, c.676A > G, c.748T > G, and c.801C > G—had been previously reported in earlier studies [20,21,22,23,24,25]. The alternative T allele of c.183C > T exhibits high frequency (0.94–1.00) in Maltese goats from Italy and Murciano-Granadina goats from Spain, but remains low (0.03–0.06) in Swiss breeds such as Camosciata delle Alpi and Saanen goats, indicating the considerable variability of allele frequencies among European breeds [20]. In contrast, the T allele frequency in Asian populations was relatively consistent, ranging from 0.33 to 0.66 across the four populations analyzed here, with similar moderate frequencies in Macheng Black goats (0.65), Ganjam and Keonjhar goats (0.51), and other populations [24,25]. Similarly, the c.748T > G variant exhibited allele frequencies comparable to those of c.183C > T across most populations [20,24,25], suggesting strong linkage disequilibrium between these two sites within each population. The G allele of c.676A > G was found at relatively low frequencies (0.09–0.43) in both European and Asian populations, except in the Macheng Black goat population, where it was observed at a frequency of 0.43. Finally, the G allele of c.801C > G showed relatively high frequencies (0.58–0.76) in Maltese and Murciano-Granadina Black goats but was detected at much lower frequencies (0.00–0.28) in breeds such as Boer, Saanen, and Ganjam goats [20].

Based on these allele frequency comparisons, c.183C > T and c.748T > G appear to be the most informative variants and may be valuable for understanding the genetic characteristics of different goat populations. Among the four nucleotide variants identified, three (c.676A > G, c.748T > G, and c.801C > G) result in amino acid substitutions. The c.676A > G variant (p.K226E) is located within the third intracellular loop (IL3) of the MC1R protein, where residues 226–229 play a critical role in G protein coupling. A mutation at position K226 has been shown to severely impair signal transduction [29]. In Boer goats, all individuals exhibiting brown coloration on the head and neck were of the AA genotype, whereas all F1 progeny resulting from crosses between Boer goats and Tangshan Dairy goats (which display white heads and carry the GG genotype) exhibited white heads and necks, suggesting that the G allele may contribute to white head coloration [22]. Moreover, the majority of individuals from 11 Chinese indigenous goat breeds with fully white coats were homozygous for the GG genotype [22]. These findings indicate that the c.676A > G variant may play a role in influencing white coat color in goats, although strong additional coat color regulatory mechanisms likely exist in other breeds.

The c.748T > G variant, located within the sixth transmembrane domain of MC1R, is critical for ligand binding and has been reported to affect basal cAMP levels and pERK1/2 signaling [24,30]. The evolutionary conservation of this region suggests that mutations at this site could have significant functional consequences [20]. Moreover, high frequencies of the G allele have been observed in Boer goats and Boer × Macheng Black hybrids, particularly among individuals with predominantly white body hair [24], suggesting that the c.748T > G variant may contribute to the regulation of white coat coloration.

The c.801C > G variant affects the cysteine residue at position 267, which forms a disulfide bond with the cysteine at position 275—an interaction critical for maintaining the three-dimensional structure of MC1R. This variant has been reported to impair MC1R function [31]. Similar to c.748T > G, it influences basal cAMP levels and pERK1/2 signaling pathways [20,24]. In Murciano-Granadina goats, individuals with solid brown coats were homozygous for the CC genotype, whereas only GG or CG genotypes were observed in goats with solid black coats [20]. Comparable findings were reported by Guan et al. [21], suggesting that the G allele promotes eumelanin synthesis in a dominant manner. However, the G allele does not fully dictate coat color, as individuals with solid red or white coats in the Derivata di Siria and Saanen breeds also carried the G allele [20].

In the present study, statistical analysis of genotype frequencies for the three amino acid-altering variants (c.676A > G, c.748T > G, and c.801C > G) revealed significant differences between the black and white and black and brown coat color groups (*p* < 0.05 for all variants). A trend toward significance was also observed for c.676A > G between the brown and white coat groups (*p* < 0.10). These results should be interpreted cautiously, given the relatively small sample size, which may have inflated the statistical significance. In particular, effective sample size would affect the statistical significance in association analysis [32]. An adequate sample size—more specifically, an adequate effective sample size—is necessary to accurately assess the association between genetic variants and traits. Nonetheless, the functional importance of c.676A > G [29] and its observed association with coat color suggests a potential role in pigmentation regulation. Similarly, the results for c.801C > G are consistent with previous findings [20,21], supporting the involvement of the G allele in coat color determination, particularly in distinguishing black from brown and white phenotypes.

## 5. Conclusions

In this study, the nucleotide sequences of the *MC1R* gene were analyzed in goats from four Asian countries (Nepal, the Philippines, Cambodia, and Kazakhstan), leading to the identification of seven genotypic variants, including three novel variants. Four of these variants had previously been reported in other goat breeds. Notably, the c.183C > T and c.748T > G variants exhibited significant allele frequency variation among populations, indicating their potential utility for distinguishing the genetic characteristics of different goat populations. Additionally, the c.147delG variant, identified specifically in Nepalese goats in the context of this research, along with the c.676A > G and c.801C > G variants, observed across multiple populations, suggests that these variants may influence MC1R’s function in Asian goat breeds. Overall, the findings of this study enhance the understanding of the genetic factors involved in coat color determination and highlight the genetic diversity underlying coat color mechanisms among different Asian goat populations.

## Figures and Tables

**Table 1:** Variants within the coding sequence of the caprine *MC1R* gene detected in the present and previous studies.

Position *	Variant	Codon	Amino Acid Substitution	SNP ID	References
16105011	c.26 G > T	CGG/CTG	p. R9L	-	[22]
16105130	c.145 G > T	GGG/TGG	p. G49W	-	[22]
16105132	c.147 delG	GGG/GG	p. L50fs	-	present study
16105168	c.183 C > T	GCC/GCT	p.61A	-	present study, [20,24,25]
16105227	c.242 C > T	GCC/GTC	p. A81V	rs646332477	[20]
16105317	c.332 C > G	GCC/GGC	p. A111G	-	[25]
16105344	c.359 T > C	ATT/ACT	p. I120T	-	present study
16105658	c.673 C > T	CAG/TAG	p. Q225X	-	[20,23]
16105661	c.676 A > G	AAG/GAG	p. K226E	rs664274065	present study, [22,23,24]
16105686	c.701 G > A	GGC/GAC	p. G234D	rs644488799	[22]
16105733	c.748 T > G	TTC/GTC	p. F250V	rs657434682	present study, [20,24,25]
16105749	c.764 G > A	GGC/GAC	p. G255D	-	[23,24]
16105760	c.775 C > T	CTG/TTG	p.259L	-	present study
16105778	c.793 G > A	GTC/ATC	p. V265I	-	[24]
16105786	c.801 C > G	TGC/TGG	p. C267W	rs669694251	present study, [20,21,24,25]

* The position on caprine chromosome 18 according to NC_030825.1.

**Table 2:** Genetic frequencies of seven variants detected in native Asian goats.

Variants		Nepal (*n* = 30)		Philippines (*n* = 30)		Cambodia (*n* = 30)		Kazakhstan (*n* = 30)		Merged (*n* = 120)	
		Freq.	n	Freq.	n	Freq.	n	Freq.	n	Freq.	n
c.147	GG	0.83	25	1.00	30	1.00	30	1.00	30	0.96	115
(delG)	G-	0.13	4	0.00	0	0.00	0	0.00	0	0.03	4
	--	0.03	1	0.00	0	0.00	0	0.00	0	0.01	1
	G	0.72		1.00		1.00		1.00		0.98	
	-	0.28		0.00		0.00		0.00		0.02	
c.183	TT	0.47	14	0.67	20	0.20	6	0.07	2	0.35	42
(T/C)	TC	0.40	12	0.17	5	0.53	16	0.53	16	0.41	49
	CC	0.13	4	0.17	5	0.27	8	0.40	12	0.24	29
	T	0.66		0.75		0.44		0.33		0.55	
	C	0.33		0.25		0.56		0.67		0.45	
c.359	TT	1.00	30	0.97	29	1.00	30	1.00	30	0.99	119
(T/C)	TC	0.00	0	0.00	0	0.00	0	0.00	0	0.00	0
	CC	0.00	0	0.03	1	0.00	0	0.00	0	0.01	1
	T	1.00		0.97		1.00		1.00		0.99	
	C	0		0.03		1.00		1.00		0.01	
c.676	AA	0.53	16	0.90	27	0.43	13	0.33	10	0.55	66
(A/G)	AG	0.37	11	0.03	1	0.37	11	0.50	15	0.32	38
	GG	0.10	3	0.07	2	0.20	6	0.17	5	0.13	16
	A	0.72		0.92		0.66		0.58		0.71	
	G	0.28		0.08		0.34		0.42		0.29	
c.748	TT	0.20	6	0.17	5	0.27	8	0.40	12	0.26	31
(T/G)	TG	0.40	12	0.17	5	0.53	16	0.53	16	0.41	49
	GG	0.40	12	0.67	20	0.20	6	0.07	2	0.33	40
	T	0.40		0.25		0.56		0.67		0.46	
	G	0.60		0.75		0.44		0.33		0.54	
c.775	CC	1.00	30	0.97	29	1.00	30	1.00	30	0.99	119
(C/T)	CT	0.00	0	0.00	0	0.00	0	0.00	0	0.00	0
	TT	0.00	0	0.03	1	0.00	0	0.00	0	0.01	1
	C	1.00		0.97		1.00		1.00		0.99	
	T	0.00		0.03		0.00		0.00		0.01	
c.801	CC	0.80	24	0.97	29	0.77	23	0.87	26	0.85	102
(C/G)	CG	0.20	6	0.03	1	0.23	7	0.10	3	0.14	17
	GG	0.00	0	0.00	0	0.00	0	0.03	1	0.01	1
	C	0.90		0.98		0.91		0.92		0.92	
	G	0.10		0.02		0.09		0.08		0.08	

Freq.: genotype and allele frequencies. Merged: a population in which all individuals of the four countries were included.

**Table 3:** Gene frequencies of country-specific variants.

	Nepal (*n* = 122)			Philippines (*n* = 110)					
	c.147 (delG)			c.359 (T/C)			c.775 (C/T)		
Genotype frequency	GG	0.88	107	TT	0.95	105	CC	0.95	105
	G-	0.10	12	TC	0.04	4	CT	0.04	4
	--	0.02	3	CC	0.01	1	TT	0.01	1
Allele frequency	G	0.93		T	0.97		C	0.97	
	-	0.07		C	0.03		T	0.03	

**Table 4:** Association between three missense variants and coat colors.

Population	Coat Color	*n*	c.676A > G			c.748T > G			c.801C > G		
			AA	AG	GG	TT	TG	GG	CC	CG	GG
Nepal (*n* = 30)	Black	17	10	5	2	2	7	8	12	5	0
	Brown	7	3	4	0	3	3	1	7	0	0
	White	6	3	2	1	1	2	3	5	1	0
Philippines (*n* = 30)	Black	17	17	0	0	1	3	13	16	1	0
	Brown	9	8	0	1	2	0	9	9	0	0
	White	4	2	1	1	2	2	0	4	0	0
Cambodia (*n* = 30)	Black	21	11	7	3	5	11	5	16	5	0
	Brown	5	2	2	1	1	3	1	3	2	0
	White	4	0	2	2	2	2	0	4	0	0
Kazakhstan (*n* = 30)	Black	21	8	9	4	8	11	2	20	0	1
	Brown	4	2	1	1	1	3	0	2	2	0
	White	5	0	5	0	3	2	0	5	0	0
Merged (*n* = 120)	Black	76	46	21	9	16	32	28	64	11	1
			(0.61)	(0.28)	(0.11)	(0.21)	(0.42)	(0.37)	(0.84)	(0.14)	(0.01)
	Brown	25	15	7	3	7	9	11	21	4	0
			(0.60)	(0.28)	(0.12)	(0.28)	(0.36)	(0.44)	(0.84)	(0.16)	(0.00)
	White	19	5	10	4	8	8	3	18	1	0
			(0.26)	(0.53)	(0.21)	(0.42)	(0.42)	(0.16)	(0.95)	(0.05)	(0.00)
*p*-value	Black vs. Brown		0.097 ^†^			0.077 ^†^			0.084 ^†^		
	Black vs. White		0.001 *			0.002 *			0.005 *		
	Brown vs. White		0.084 ^†^			0.309			0.267		
	Black vs. Others		0.189			0.547			0.935		

Merged: A population including all individuals from the four countries. * and ^†^ indicate significant (*p* < 0.05) and tendency-level (*p* < 0.10) differences in genotype frequencies, respectively.

## Data Availability

The original data presented in the study are openly available in DDBJ at LC881802-LC881921.

## Supplementary Materials

The following supporting information can be downloaded at https://www.mdpi.com/article/10.3390/ani15142026/s1, Figure S1: Mechanism of coat color detection; Figure S2: Coat colors for Nepal goats with each genotype of c.147delG; Figure S3: Coat colors for Philippines goats with each genotype of c.359T < C and c.775C < T; Figure S4: Estimated MC1R protein structure with I or T amino acid at p.I120T; Table S1: Sample detail in Asian goats.

## Abbreviations

The following abbreviations are used in this manuscript:

MC1R	Melanocortin 1 receptor
α-MSH	α-melanocyte-stimulating hormone
CDS	Coding sequence
RSMD	Root mean square deviation
TM	Transmembrane domain
EL	Extracellular loop
IL	Intracellular loop
cAMP	Cyclic adenosine monophosphate
pERK	Phosphorylated extracellular signal regulated kinase
