# Supplementary material for: Detection of MC1R Genetic Variants and Their Association with Coat Color in Asian Goats
Source: Animals (Basel). 2025 Jul 10;15(14):2026. doi: 10.3390/ani15142026 (PMC12291834; doi:10.3390/ani15142026)
Supplement: Supplementary file 1 [file animals-15-02026-s001.zip › animals-3650515_FigS_ver.2-Animals.pdf]

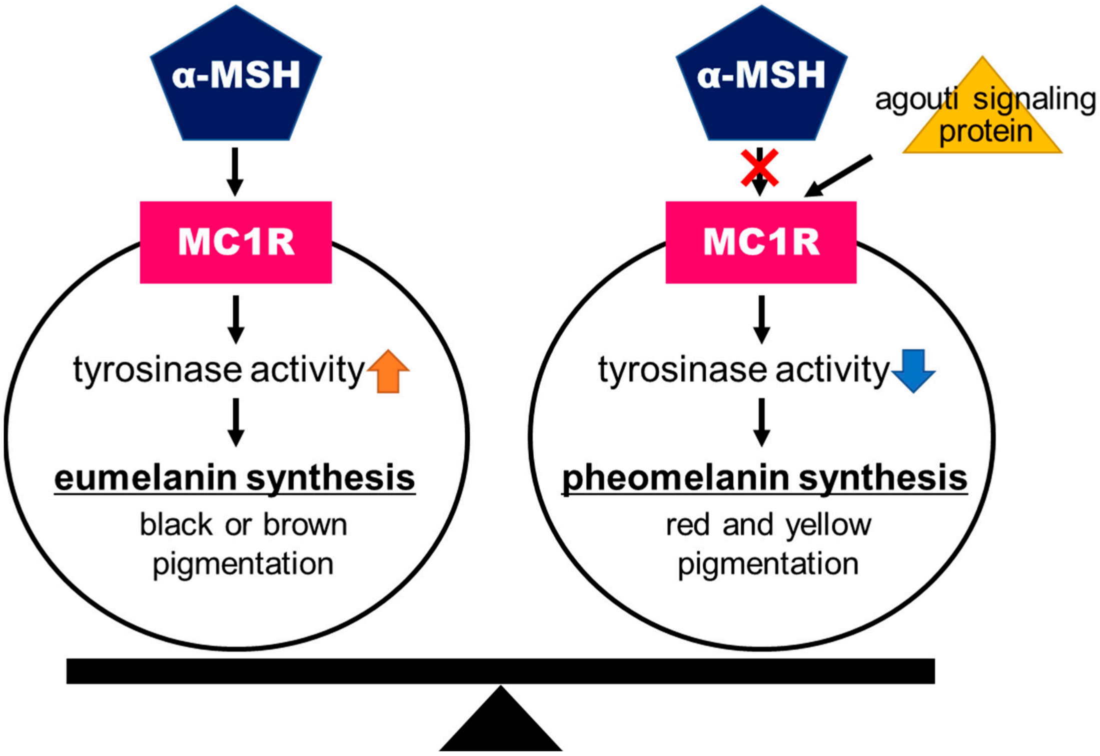

Figure S1. Mechanism of coat color detection. The coat color is determined by the ratio of eumelanin and pheomelanin synthesis.

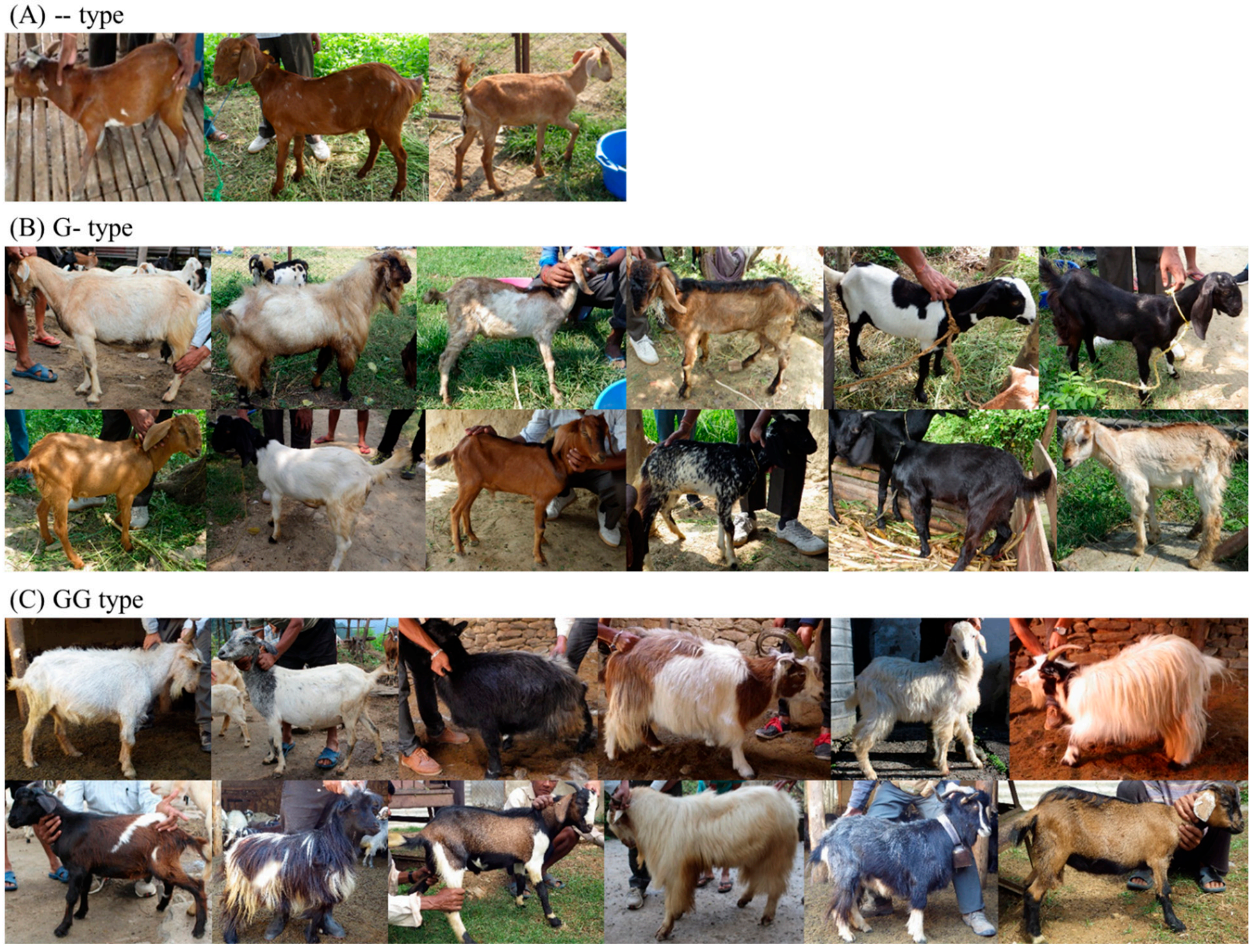

Figure S2. Coat colors for Nepal goats with each genotype of c.147delG. A: -- type goats (n = 3), B: G- type goats (n = 12) C: GG type goats (n = 107, randomly selected).

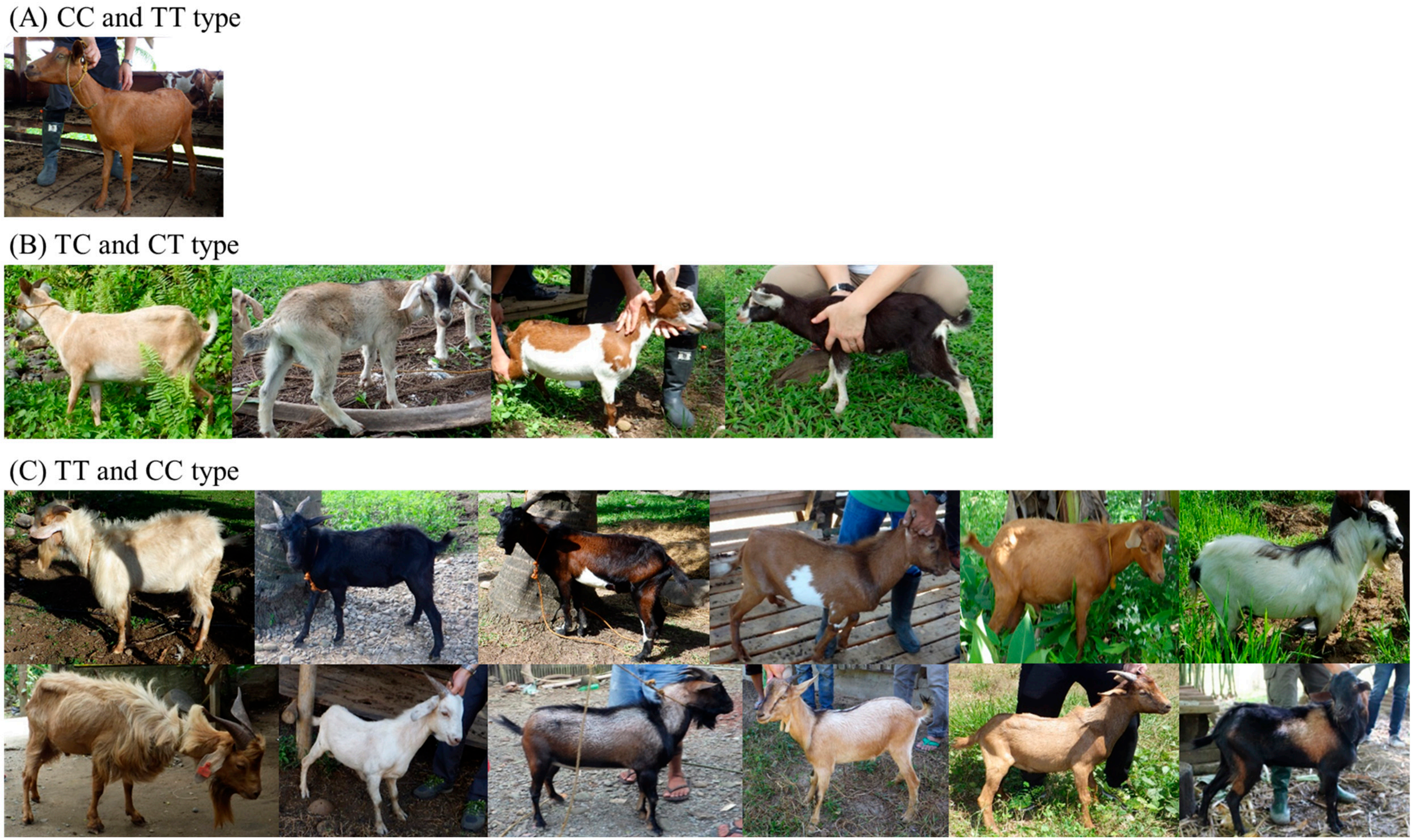

Figure S3. Coat colors for Philippines goats with each genotype of c.359T<C and c.775C<T. A: CC and TT type goats (n = 1), B: TC and CT type goats (n = 4) C: TT and CC type goats (n = 105, randomly selected).

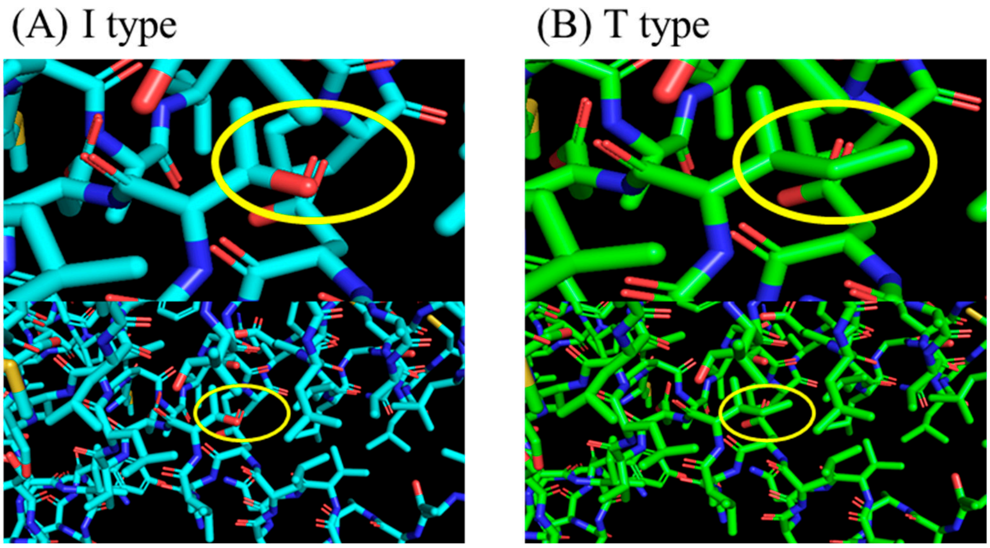

Figure S4. Estimated MC1R protein structure with I or T amino acid at p.I120T. The yellow circle represents the position of p.I120T.
